# The Role of S-Nitrosylation and S-Glutathionylation of Protein Disulphide Isomerase in Protein Misfolding and Neurodegeneration

**DOI:** 10.1155/2013/797914

**Published:** 2013-11-18

**Authors:** M. Halloran, S. Parakh, J. D. Atkin

**Affiliations:** ^1^Department of Neuroscience in the School of Psychological Science, La Trobe University, Bundoora, VIC 3086, Australia; ^2^Department of Biochemistry, La Trobe University, Bundoora, VIC 3086, Australia

## Abstract

Neurodegenerative diseases involve the progressive loss of neurons, and a pathological hallmark is the presence of abnormal inclusions containing misfolded proteins. Although the precise molecular mechanisms triggering neurodegeneration remain unclear, endoplasmic reticulum (ER) stress, elevated oxidative and nitrosative stress, and protein misfolding are important features in pathogenesis. Protein disulphide isomerase (PDI) is the prototype of a family of molecular chaperones and foldases upregulated during ER stress that are increasingly implicated in neurodegenerative diseases. PDI catalyzes the rearrangement and formation of disulphide bonds, thus facilitating protein folding, and in neurodegeneration may act to ameliorate the burden of protein misfolding. However, an aberrant posttranslational modification of PDI, S-nitrosylation, inhibits its protective function in these conditions. S-nitrosylation is a redox-mediated modification that regulates protein function by covalent addition of nitric oxide- (NO-) containing groups to cysteine residues. Here, we discuss the evidence for abnormal S-nitrosylation of PDI (SNO-PDI) in neurodegeneration and how this may be linked to another aberrant modification of PDI, S-glutathionylation. Understanding the role of aberrant S-nitrosylation/S-glutathionylation of PDI in the pathogenesis of neurodegenerative diseases may provide insights into novel therapeutic interventions in the future.

## 1. Introduction

Neurodegenerative diseases share several common pathological characteristics, including the aberrant aggregation of misfolded proteins, leading to the formation of abnormal protein inclusions [1]. These diseases are also frequently classified as protein conformational disorders in which protein aggregation occurs due to the exposure of hydrophobic regions [2]. The most common neurodegenerative diseases include Alzheimer's disease (AD), Parkinson's disease (PD), amyotrophic lateral sclerosis (ALS), Creutzfeldt-Jakob disease (CJD), and Huntington's disease (HD). These diseases differ according to the specific group of neurons targeted and the type of misfolded proteins that aggregate. In AD, the accumulation of aggregated proteins occurs in cortical regions and involves both *β*-amyloid (*β*A), which forms extracellular amyloid plaques, and tau, which is hyperphosphorylated and forms intracellular neurofibrillary tangles (NFT) [3, 4]. PD involves the formation of Lewy bodies (LB) containing misfolded *α*-synuclein [5], and in HD aggregated Huntington protein with expanded polyglutamine repeats forms inclusions in the nucleus [6]. Similarly, in ALS, cytoplasmic inclusions contain copper/zinc (CuZn) superoxide dismutase 1 (SOD1) [7–9], TAR DNA binding protein 43 (TDP-43) [10–13], or fused in sarcoma/translated in liposarcoma (FUS/TLS) [14]. Recently, a hexanucleotide repeat expansion in an intronic region of the chromosome 9 open reading frame 72 (C9orf72) gene, encoding a gene of unknown function, was linked to the greatest proportion of familial ALS cases [15, 16]. For AD, PD, and ALS, 90–95% of cases arise sporadically, while the remainder are familial in nature. Genetic mutations in Amyloid Precursor Protein (APP), leads to increased accumulation of A-*β* and fibril formation [17–20], and Presenilin 1, 2 (PS 1, 2), which regulates APP processing via gamma secretase [21–23], causes rare familial cases of AD [24]. Similarly, some forms of autosomal dominant familial PD is caused by *α*-synuclein mutations [25] leading to the aggregation of *α*-synuclein into insoluble fibrils, which are the primary components of LB [26], while mutations in PINK1, Parkin, and DJ-1 cause autosomal recessive PD cases [27]. However, in contrast to these conditions, HD is early onset and entirely genetic in nature.

The causal factors underlying the pathogenesis of sporadic neurodegenerative diseases remain poorly understood. However, due to the typical late onset of these disorders, neurodegeneration can be conceptualized as pathology that arises during the normal aging process, involving increases in oxidative stress and the production of free radicals which damage cells by decreasing antioxidant defenses. In AD, increased free radical accumulation and elevated levels of oxidative and nitrosative stress are associated with alterations in A-*β* metabolism [28, 29]. Meanwhile, in PD, nitrosative stress is associated with impairment of the mitochondrial respiratory chain, leading to energy deficiency and cell death [30]. In addition, oxidative and nitrosative stress are associated with endoplasmic reticulum (ER) stress, through the accumulation of misfolded proteins in the ER, and upregulation of molecular chaperones in the protein disulphide isomerase (PDI) family [31]. PDI possesses both general protein chaperone and disulphide interchange activity, thus facilitating the formation of native disulphide bonds in proteins. It also facilitates the degradation of these proteins via ER-associated degradation (ERAD), whereby irreparably misfolded proteins are targeted for retrotranslocation to the cytoplasm, where they undergo polyubiquitination and subsequent degradation by the proteasome [32–35]. There is now sufficient evidence that in conditions of elevated nitrosative stress, PDI undergoes an aberrant posttranslational modification known as S-nitrosylation, which inhibits its enzymatic activity [36]. Hence, in late onset neurodegenerative disease, there is a decrease in cellular defences and a corresponding increase in oxidative and nitrosative damage to lipids, proteins, DNA, and RNA [37, 38].

In this review, we will begin by examining the role of nitrosative stress, redox potential, and S-nitrosylation/S-glutathionylation of proteins linked to neurodegeneration. The structure and function of PDI family members will be discussed, and the importance of PDI in neurodegenerative disease will be highlighted. We will examine the evidence that PDI is aberrantly S-nitrosylated and discuss the functional significance of this modification in neurodegeneration. Finally, we speculate that PDI may also be S-glutathionylated in neurodegenerative disease.

## 2. Nitrosative Stress

Reactive nitrogen and oxygen species (RNS and ROS), primarily superoxide anion (O_2_^−^), hydrogen peroxide (H_2_O_2_), or nitric oxide (NO), are highly reactive molecules that normally function at low levels as mediators of intracellular signalling processes in mammalian cells [36, 39]. However, RNS and ROS can accumulate in cells under pathological conditions, triggering nitrosative or oxidative stress. This leads to numerous detrimental effects on cellular function including posttranslational modifications of proteins, lipid peroxidation, DNA, damage, and dysregulation of redox signalling [28, 37, 38, 40]. Nitrosative or oxidative stress results when there is an imbalance between the production of RNS/ROS and cellular antioxidant defence mechanisms such ascorbic acid, glutathione (GSH), or enzymes including superoxide dismutases, catalases, and glutathione peroxidases. GSH is a particularly important antioxidant as it is the most abundant cellular thiol-containing molecule; the ratio of reduced GSH to its oxidized form (GSSG) makes a major contribution to cellular redox potential and homeostasis [28, 29, 41]. However, the thiol/disulfide systems, which include GSH/GSSG, and plasma cysteine/cystine (Cys/CySS) pools are not necessarily in equilibrium and may respond differentially to specific stressors [42]. Nitrosative or oxidative stress may be induced by familial mutations, exogenous toxins (xenobiotics, pesticides), or via normal aging processes such as alterations in mitochondrial respiration [31, 43]. Neurons are particularly vulnerable to the effects of RNS/ROS due to a relative deficiency in antioxidant enzymes glutathione peroxidase (GPx) and catalase (Cat), compared to other cell types, and their higher metabolic demands which generate RNS/ROS from mitochondrial metabolism [38, 39, 43, 44].

RNS are derived primarily from O_2_^−^ and NO, a small, diffusible inter- and intracellular messenger that normally mediates many intracellular signalling pathways [29, 31, 45, 46]. NO is generated by NO synthases (NOS) that use oxygen (O_2_) and nicotinamide adenine dinucleotide phosphate (NADPH) oxidase to convert L-arginine to L-citrulline [47]. NOS is constitutively expressed in several isoforms in the central nervous system (CNS): endothelial NOS (eNOS), inducible NOS (iNOS), neuronal NOS (nNOS), and an isoform expressed in the inner mitochondrial membrane (mtNOS) [48–50]. The covalent addition of NO to a cysteine thiol or thiolate anion on specific proteins, to form an S-nitrosothiol (SNO) group, is a process termed “S-nitrosylation” [36, 51–56].

## 3. S-Nitrosylation

In recent years, S-nitrosylation has been increasingly implicated in many physiological and pathological conditions [36]. Under normal conditions, S-nitrosylation is a reversible posttranslational modification analogous to acetylation and phosphorylation that regulates protein activity [55, 57]. The SNO-group can be removed in these situations by denitrosylation enzymes, primarily S-glutathione reductase (GSNOR; alcohol dehydrogenase III) in conjunction with GSH and NADH as an electron donor [58, 59]. However, reduced oxidoreductase thioredoxin (TRX) [60, 61] can oxidize S-nitrosoglutathione (GSNO) to release GSH and NO [62, 63]. Recombinant human PDI can denitrosylate GSNO [64] and *in vitro* SOD1 can modify the stability of S-nitrosothiols by enhancing the decomposition of GSNO, resulting in production of NO [65], possibly by its reduced metal ions [66].

S-nitrosylation is both a reversible and irreversible process [67]. Under pathological conditions, S-nitrosylation of specific proteins is an abnormal, irreversible process and is linked to protein misfolding, ER stress, mitochondrial dysfunction, synaptic degeneration, and cell death [36]. A well-recognized mechanism for NO production in neurodegenerative diseases is activation of N-methyl-D-Aspartate receptors (NMDAr) [68, 69]. Activation of NMDArs generates ROS and results in calcium (Ca^2+^) influx into the cell [31, 70–72], which in turn activates nNOS to produce NO [50]. S-nitrosylation may also lead to NO-independent oxidation of proteins via ROS, producing reversible modifications in the form of intramolecular/mixed disulphide bonds. One of the proposed pathways for the further oxidation of cysteines is through the hydrolysis of sulfenic acid (SOH), which then may be susceptible to irreversible oxidation from accumulating ROS leading to stable sulfinic (–SO_2_H) or sulfonic (–SO_3_H) acid formation [73–75]. However, –SO_2_H can be reduced back to the free thiol group if the enzyme sulfiredoxin is induced and this can occur in neurons due to activation of NMDAr by increased synaptic activity [76]. In addition, S-nitrosylation can reversibly influence further posttranslation modifications of cysteine residues. When there are two proximal cysteine residues, S-nitrosylation of one of these can facilitate disulphide bond formation [77–79]. Under conditions of excessive nitrosative stress, however, S-nitrosylation inhibits the formation of disulphide bonds [67, 75]. Another pathological mechanism linked to S-nitrosylation has also been implicated in ALS. Cells expressing familial ALS mutants, SOD^A4V^ and SOD^G37R^, have increased denitrosylation activity of GSNO in comparison to wild type (WT) SOD1 [80]. This deficiency in S-nitrosylation is especially elevated in mitochondria of mutant SOD1 cells [81].

Whilst most proteins contain multiple cysteine residues, the features underlying the specificity for S-nitrosylation are not fully defined, but appear to rely on tertiary rather than primary structure. Previous studies have suggested that the formation of S-nitrosylated proteins (SNO proteins) requires a cysteine flanked by a proximal acid-base motif, hydrophobic content, low pKa, and high exposure of sulfur atoms [67, 82]. However, a recent bioinformatics study predicted that the known SNO-Cys sites in proteins are more heterogenous than this, although the presence of a charged residue in close proximity to NO-Cys and another oppositely charged residue within a larger region was a common feature [82]. The stability of the resulting SNO-group depends upon the local environment of the cysteine residues, but studies of the dissociation energies of the S–N bond suggest that there is a wide variation, with this bond remaining stable theoretically from seconds to years [83, 84].

Up to one thousand SNO proteins have now been identified [85] including many proteins linked to neurodegenerative diseases [36, 77, 86–89]. For instance, S-nitrosylation of dynamin-related protein (Drp1) (SNO-Drp), found in post-mortem brains of AD cases, is associated with *β*-A for-mation and subsequent activation of mitochondrial fission [77, 87]. In sporadic and familial PD, S-nitrosylated Parkin (SNO-Parkin) has reduced E3 ligase function, leading to proteasomal dysfunction [90]. Similarly, proteins involved in apoptosis (XIAP/Caspase 3, GAPDH-Siah), antioxidant activity (Prx2), the phosphatase pathway (PTEN), neuroinflammation (COX2), and autophagy (JNK1 and IKK*β*) are also S-nitrosylated (for comprehensive review see [36]). Furthermore, SNO-proteins may alter cellular redox homeostasis through an interaction with GSH and therefore may influence other post-translational modifications, such as S-glutathionylation [36, 41]. Some proteins, such as NMDAr, are S-nitrosylated under both normal and pathological conditions [36]. S-nitrosylation/denitrosylation of NMDAr is important in physiological cellular signalling processes [52, 53, 91], but overactivation is associated with an increased production of SNO-proteins and neurodegeneration [31]. However, it should be noted that S-nitrosylation of NMDAr at Cys399 is protective by deactivation of the receptor, thus preventing glutamate excitotoxicity [53, 67, 78, 91].

## 4. S-Glutathionylation

S-glutathionylation is another posttranslational modification that has been implicated in the regulation of diverse proteins involved in energy metabolism, signalling pathways, Ca^2+^ homeostasis, antioxidant enzymatic activity, and protein folding [92] (for a comprehensive review see [41]). S-glutathionylation is induced by RNS/ROS and involves the formation of a disulfide between GSH and a cysteine residue [41]. As reduced GSH is the most abundant cellular thiol, it plays an important role in S-glutathionylation [41], although protein thiols represent a similar redox pool, and therefore may also be critical in providing antioxidant protection against oxidative stress [93]. S-nitrosylated cysteines can be converted to S-glutathionylated cysteines, supporting the premise that products of nitrosative stress induce S-glutathionylation [41]. However, the exact identity of the metabolites that act as proximal donors in this reaction remain to be elucidated [41] and it is unclear whether SNO proteins are intermediates for S-glutathionylation *in vivo*. Under oxidizing conditions, S-glutathionylation is reversible via the release of GSH from cysteine residues by thiol-disulphide oxidoreductase enzymes (TDOR). TDOR enzymes include TRX, which reduces intra- and intermolecular disulphide bonds, and glutaredoxin (GRX) which reduces protein-GSH bonds [94–96]. TRX and GRX catalyze the reduction of disulphide bonds and reactivate proteins that have undergone oxidation from sulfhydryl groups [95, 96]. Alterations in the ratio of GSH/GSSG and conditions that promote RNS/ROS production result in cysteine modifications that are precursors to the formation of mixed disulphides with GSH [95, 97, 98]. However, the role of S-glutathionylation during nitrosative and oxidative stress has not been completely defined. Glutathionylation at physiological levels may therefore represent a mechanism whereby cysteine residues faced with oxidation are protected from irreversible damage. The reduction of GSH-protein disulphide by GRX is essential in this process as it maintains the cellular availability of GSH and acts in concert with TRX to maintain the cellular thiol status [95].

S-glutathionylation has been implicated in neurodegeneration [95, 99–101]. The ratio of GSH/GSSG decreases in brains of aged rats [102], and accumulation of S-glutathionylated p53 in the inferior parietal lobule of AD patients has also been reported [101]. In PD models, administration of the neurotoxin 1-methyl-4-phenyl-1,2,3,6-tetrahydropyridine (MPTP), which causes damage to dopaminergic neurons, caused an early decrease in the levels of GSH, inhibition of mitochondrial complex 1, and dopaminergic cell loss [103]. Furthermore, increases in GSH, GRX, and GSH reductase were detected in the brains of transgenic HD mice models (R6) [104, 105]. S-glutathionylation of SOD1 isolated from human erythrocytes at Cys111 promoted SOD1 monomer formation and subsequent aggregation [106]. Hence, alterations in S-glutathionylation and redox potential are important mediators of protein misfolding, and aberrant disulphide bond formation is implicated in this process.

## 5. ER Stress and Neurodegeneration

The major cellular location for protein disulphide bond formation is the ER. The highly oxidizing environment of this compartment (GSH : GSSG ratio~3 : 1) is necessary for formation of disulphide bonds and is in stark contrast to the reducing environment of the cytosol (GSH : GSSG ratio~100 : 1) [41, 92, 107]. The ER environment, therefore, is highly sensitive to changes in nitrosative and oxidative stress [31, 36].

ER stress is increasingly implicated as a pathogenic mechanism in neurodegenerative diseases [108–114]. ER stress occurs when misfolded proteins accumulate within the ER lumen, triggering the unfolded protein response (UPR) [115]. The UPR is a set of signalling pathways that initially aim to restore homeostasis by: (1) reducing protein synthesis and translocation, attenuating further accumulation of unfolded proteins in the ER, (2) activation of ER-resident chaperones including PDI to increase the protein folding capacity of the ER, and (3) induction of ERAD. The UPR activates three ER stress sensor proteins: inositol requiring kinase 1 (IRE1 *α*/*β*), double-stranded RNA-activated protein kinase- (PKR-) like ER kinase (PERK), and activating transcription factor 6 (ATF6), which transduce signals to the nucleus and cytosol [115, 116]. However, if homeostasis cannot be restored, apoptosis is triggered [115, 117]. Prolonged UPR activation linked to RNS or ROS triggers apoptosis through C/EBP homologous protein (CHOP), caspase 4, c-Jun, and c-Jun N-terminal kinase (JNK) [41, 118, 119]. 

PDI family members fulfil crucial roles in regulating ER stress by maintaining native protein conformation and facilitating protein degradation [120]. The remainder of this review will focus on the PDI family and the effect of S-nitrosylation/S-glutathionylation on PDI and its functional role in neurodegeneration.

## 6. PDI Family Members

There are currently 21 identified members of the PDI family [32, 120–125], which share several features in common; at least one domain with a TRX fold, the presence of a signal sequence, and ER localization due to the presence of an KDEL or other ER retention signal [32, 120, 126]. Whilst PDI family members contain a TRX domain, they essentially differ from TRX due to their higher redox potentials, substrate binding domains, and their ability to display both isomerase and chaperone activities, which renders them more efficient than TRX at forming/reforming disulphide bonds [127, 128]. Whilst PDI family members primarily mediate protein folding, other functions have also been ascribed to them, including regulation of Ca^2+^ homeostasis [129, 130] and ERAD, thus ameliorating protein misfolding within the ER [33–35].

PDI disulphide-isomerase activity catalyzes the rearrangement of nonnative (incorrectly formed) disulphide bonds on nascent proteins, which would otherwise result in the formation of a misfolded structure. This activity is mediated through catalysis of thiol disulphide exchange (isomerization), whereby non-native disulphide bonds are initially reduced, and then oxidized to form the native structure [131–133]. Disulphide formation and stability are facilitated by the redox conditions of the ER [31]. Thus, active-site cysteines shift between two redox states: oxidation and the formation of disulphide bonds and reduction leading to the formation of thiols with free sulfhydryls [134]. In addition, PDI also has general chaperone activity which is independent of its disulphide interchange function [135–137]. This chaperone activity does not require its catalytic domains or active sites [138, 139].

**Figure 1:**
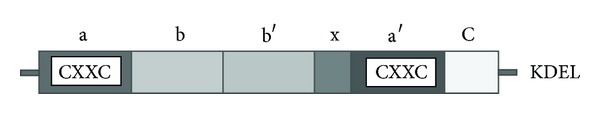
Domains of PDIA1. TRX-like domains representing catalytic active domains a a′. The b domain and b′ are catalytically inactive. The linker region is responsible for binding to the substrate. The C terminal is followed by an ER retrieval signal KDEL.

PDI (PDIA1), the prototype of the PDI family, is a 55 kDa protein with two catalytically inactive TRX domains (b and b′), inserted between two TRX-like catalytic domains (a and a′), and an acidic C terminal domain with an ER-retention motif (KDEL). PDIA1 contains a CXXC catalytically active motif (Figure 1). All domains of PDI are required for efficient catalysis of disulphide bond formation and rearrangement [32, 120, 140]. The structure of yeast PDI has revealed that the binding of PDI to misfolded protein substrates is facilitated by two of the active cysteines positioned on opposite sides of the molecule [140, 141]. The noncatalytic b′ domain is situated on the base and is the major site for binding of substrates [141], although other domains also contribute to this process. The b-b combination of noncatalytic domains is present only in PDIA1, PDIA2 (PDIp), PDIA3 (ERp57), and PDIA4 (ERp72) family members [142–146]. PDIA1 has the broadest substrate specificity of the PDI family members examined to date [144].

PDIA2 is primarily expressed in pancreatic cells and dopaminergic neurons [146–148]. The domain structure of PDIA2 is similar to PDIA1, with a CXXC motif in the homologous a, a′ domains, intervening b, b′ domains, a x-linker region, and an N-terminal ER sequence [149]. PDIA2 also contains a KEEL motif at the C-terminus, an ER retention signal analogous to KDEL [150]. Similar to PDI, PDIA2 can interact with protein substrates with and without cysteine residues [148, 151], suggesting that PDIA2 may act as a chaperone independent of catalyzing disulphide bond formation [147]. However, although its domain organization is similar to PDI, its physiological role remains unclear.

PDIA3 is the second most abundant soluble protein after PDIA1 found in the ER [120]. It contains a protein sequence homologous to PDIA1, with similarities in domain architecture but differences in substrate binding [152]. Whilst PDIA3 is an oxidoreductase with thiol-dependent reductase activity [153], it is different to the other PDI family members in that it acts primarily on glycosylated proteins by associating noncovalently with the lectin chaperones calnexin and calreticulin [154]. The catalytic properties differ from PDIA1 and the redox potential of PDIA3 is also lower than PDIA1 [155, 156]. PDIA3, like PDIA1, has two CXXC motifs at the conserved active sites and four similar TRX-like domains (a-b-b′-a′) [153, 156]. The C-terminus of PDIA3 has an ER retention signal with a sequence similar to that of PDIA1 [153] and a nuclear localisation signal near the C terminal with a high affinity for importin [128, 157, 158]. In addition, PDIA3 and PDIA1 differ in terms of substrate binding specificity due to differences in homology in their b′ domains. The binding domain of PDIA3 is enriched in lysine and arginine residues, so that PDIA3 binds to proteins containing negatively charged P domains, such as those found in calreticulin [142, 158]. The oxidative and catalytic property of PDIA3 and PDIA1 both rely on a charged glutamic acid and a pair of lysine residues found behind the active CXXC site [120].

Some PDI family members possess more than two CXXC active sites. PDIr, Erp46, and PDIA4, also known as Ca^2+^ binding protein (CaBP2) [159], all have three active sites [121, 160–163], and ERdJ5 contains four active sites [164]. PDIA4 is similar to PDIA1 in its catalytic domains but has lower sequence similarity in the other domains. It can also act as a substitute for PDIA3 in folding specific proteins, but it does not bind to glycoproteins [165]. Other PDI gene family members include DNAJC10, ERP27, ERP29 (ERP28), ERP44, PDIA5, PDIA6, PDILT, and TXNDC5 (for comprehensive review please refer to [125]). However, this review will focus on PDIA1, PDIA2, PDIA3, and PDIA4 as these are the only PDI family members to date that are reported to undergo S-nitrosylation.

## 7. The Presence of PDI in Non-ER Compartments

Whilst PDI family members are primarily considered to be ER-localized, they are also present in other cellular locations, including the nucleus, cytoplasm, cell surface, and extracellular space [128]. Few proteins linked to neurodegeneration are present in the ER, so it is possible that PDI plays an important role in these locations. In the ER, PDI must be maintained in a balance between its oxidized and reduced states to facilitate disulphide bond formation [166, 167]. However, in non-ER compartments, PDI family members have an increased ability to catalyze the reduction of disulphide bonds compared to the ER [168]. The mechanism of transit of PDI from the ER remains unknown, and because of the presence of the KDEL retention signal, observations of non-ER localized PDI have previously been questioned [128]. However, other primarily ER-localized proteins that possess a KDEL motif, such as calreticulin and binding immunoglobulin protein (BiP), are also secreted and located in the nucleus, cytoplasm and cell surface [169–176].

PDI in the cytosol has been postulated to act as a cofactor with insulin-degrading enzyme (IDE) during insulin metabolism, while acting in concert with reduced GSH to catalyze disulphide bond cleavage [177]. There is also evidence that PDI redistributes away from its ER location into the cytoplasm in pathological conditions. ER stress causes the redistribution of PDIA1 and PDIA3 from the ER to the cytosol [178], consistent with the notion that PDI in locations other than the ER is neuroprotective. Furthermore, one study demonstrated that overexpression of reticulon-4A (NOGO A) triggered the redistribution of PDI from the ER into vesicular-type structures localized in an undefined cellular compartment, both *in vitro* and *in vivo*, which occurred in the absence of the UPR [179]. Deletion of NOGO A, B from ALS mouse models, involving transgenic overexpression of mutant SOD1^G93A^, led to earlier onset and increased disease progression, indicating that reticulons mediate PDI function and redistribution in neurodegeneration [179]. A more recent study, using human neuroblastoma SH-SY5Y cells overexpressing reticulon protein 1C (RTN-1C), demonstrated that redistribution of PDI away from the ER into vesicular structures led to a consequent increase in the enzymatic activity of PDI and a decrease in S-nitrosylation [180].

PDI has also been detected at the cell membrane, where a role in NO signalling has been described. S-nitrosylated extracellular proteins transfer NO to the cytosol via the reducing activity of cell surface PDI [181, 182]. During this process, cell-surface PDI also undergoes thiol modification [183]. Furthermore, PDIA3 interacts with prion proteins (PrP) at the cell surface and may play a key role in PrP accumulation [184]. In addition, PDIA1 and PDIA3 have been detected in the nucleus, where they are posited to anchor DNA loops to the nuclear matrix [128, 185, 186]. PDI-like activity has also been detected in mitochondria, although PDIA1 has not been identified in this compartment [187], and it is possible that Mia 40 contributes to this activity [188, 189].

PDIA1 and PDIA3 have also been detected at mitochondrial-associated ER membranes, where, remarkably, they may regulate apoptosis signalling [190]. The expression of polyglutamine expanded Huntington protein led to PDIA1 and PDIA3 accumulation in this location, where it triggered mitochondrial outer membrane permeabilization through activation of proapoptotic BCL-2 family members, triggering apoptosis [190]. Hence, whilst PDI functions protectively through its chaperone and isomerase activities [191], it can also trigger pro-apoptotic mechanisms [190]. While this process has not yet been fully defined, the novel proapoptotic function of PDI may represent a new link between protein misfolding and cell death.

## 8. Role of PDI in Neurodegeneration

There is now substantial evidence linking PDI family members to protein misfolding in neurodegeneration. PDIA1 is upregulated in AD brain tissues [192], PDIA3 forms a complex with calreticulin and A-*β* peptides in patients' CSF [193], and NFTs are immunopositive for PDI [194, 195]. Similarly, in cellular models of PD, treatment of dopaminergic neurons with 6-hydroxydopamine (6-OHDA) induces ER stress, oxidation, and aggregation of PDIA3 [196]. PDIA2 is upregulated in SH-SY5Y human neuroblastoma treated with either 1-methy-4-phenyl-pyridinium (MPP+) or proteasome inhibitor lactacystin while immunoreactivity to PDIA2 has also been detected in LB in postmortem brains of PD patients [146]. Furthermore, the a′ domain of PDIA1 inhibits *α*-synuclein fibril formation [197], and coexpression of PDIA1 decreased synphilin-1 positive LB formation in the cytoplasm [75]. PDIA1 was upregulated in the brains of Creutzfeldt-Jakob disease (CJD) patients [198], while PDIA1 and PDIA3 were upregulated in prion disease in scrapie infected rodents [199]. Pharmacological inhibition of PDIA3 using bacitracin increased the accumulation of aggregated PrP, also suggesting that PDI is not functional in prion disease [184]. Furthermore, upregulation of PDIA1 and PDIA3 was associated with mitochondrial dysfunction in cells expressing misfolded PrP [199]. The detection of mitochondrial apoptosis triggered by PDIA1 and PDIA3 in HD models [190] also highlights the intrinsic link between PDI upregulation and mitochondrial dysregulation in neurodegeneration [199].

There is also increasing evidence for an important role for PDI in ALS. PDIA1 is upregulated and is a component of TDP-43 and FUS-positive cytoplasmic inclusions in motor neurons of sporadic ALS patients [200, 201]. Additionally, PDIA1 is a risk factor for the development of ALS [202]. PDIA1 also colocalizes with mutant SOD1-positive inclusions in cell culture and transgenic SOD1 rodents [89, 203, 204]. Overexpression of PDIA1 decreases the formation of mutant SOD1 inclusions whereas knockdown of PDI using siRNA increases the proportion of inclusions [89]. Furthermore, a synthetic mimic of the PDIA1 active site; (±)-trans-1,2-bis (mercaptoacetamido)cyclohexane (BMC), is protective against mutant SOD1 aggregation in cell culture [89]. SOD1 contains four cysteine residues, and non-native disulphide bonds between Cys6 and Cys111 have been implicated in mutant SOD1 aggregation [205]. Conversely, upregulation of PDIA1 in microglia in SOD1^G93A^ mice was associated with increased levels of NADPH oxidase (NOX), superoxide, and tumour necrosis factor-*α*. Pharmacological inhibition and knockdown of PDIA1 using siRNA decreased superoxide and NOX activation in microglia, therefore providing further evidence for a potential neurotoxic role of PDIA1 [206].

PDI is therefore upregulated during UPR activation and is part of a cellular protective mechanism that prevents protein misfolding and aggregation in neurodegeneration. PDI family members are especially vulnerable to oxidative and nitrosative-linked posttranslational modifications due to the highly oxidizing environment of the ER and the presence of cysteine residues in the PDI catalytic regions. Irreversible S-nitrosylation of PDI (SNO-PDI) may therefore ameliorate its protective function in neurodegenerative disorders and thus contribute to disease.

## 9. SNO-PDI and Neurodegeneration

PDI is S-nitrosylated by endogenous nNOS in both its TRX domains leading to a significant reduction in isomerase and chaperone activity [75]. Also, induction of SNO-PDI using NO donor S-nitrosocysteine (SNOC) completely abrogates the catalytic activity of PDI, resulting in neuronal cell death [207]. 

SNO-PDI has been detected in postmortem brain tissue of sporadic PD and AD patients [75] and lumbar spinal cord tissues of ALS patients and SOD1^G93A^ mice [89]. This was linked to excessive production of NO or exposure to exogenous agents such as rotenone [75]. PDI was shown to be modified in the cysteine thiol groups in the C-terminal CXXC motif, leading to the accumulation of polyubiquitinated proteins and activation of the UPR [75]. SNO-PDI formation is associated with synphilin misfolding in PD [31] and mitochondrial mediated apoptosis in prion infection [199]. SNO-PDI is also found in cultured astrocytes after ischemia/reperfusion-induced iNOS production, leading to increases in ubiquitinated aggregates that colocalize with SOD1 [7].

**Figure 2:**
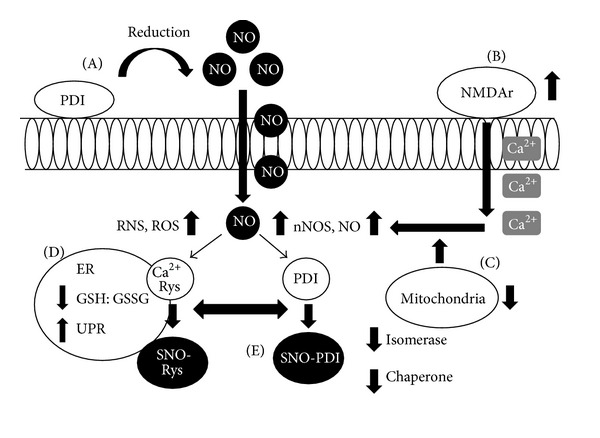
Cell surface PDI, NO, and SNO-PDI. (A) Cell surface PDI reduces NO from extracellular SNO proteins (SNO-P) and in the process undergoes thiol modification. (B) Hyperactivation of the NMDAr leads to an intracellular influx of Ca^2+^ ions (NMDAr may also undergo reversible S-nitrosylation to ameliorate excessive activity). (C) Inhibition of mitochondria contributes to an increase in intracellular NO which is potentially oxidized by O_2_ leading to an increase in NO, nNOS, ROS, and RNS. (D) Increases in RNS/ROS alters the ER redox environment, and NO S-nitrosylates Ca^2+^ ryanodine (Ryn) receptor leading to a disruption in Ca^2+^ homeostasis. (E) ER-resident proteins such as PDI are vulnerable to S-nitrosylation, deactivating its isomerase and chaperone activity, leading to accumulation of misfolded proteins, ER stress, and UPR induction.

One potential physiological mechanism of SNO-PDI production involves pathological hyperactivation of NMDAr [31] and inhibition of mitochondria leading to the generation of ROS, nNOS, and NO [31, 70, 71]. Exposure of cortical neurons to NMDA produces SNO-PDI, leading to an increase in polyubiquitinated proteins and apoptosis after 24 hrs of treatment. Furthermore, overexpression of WT PDI leads to a decrease in polyubiquitination and apoptosis, suggesting that PDI may provide protection against excitotoxicity from excessive stimulation of NMDA receptors [75]. Additionally, treatment with Rotenone, a mitochondrial complex inhibitor, produces elevated levels of SNO-PDI [75], suggesting that mitochondria are another source of NO or cytosolic nNOS [31]. NO disrupts Ca^2+^ homeostasis, potentially via S-nitrosylation of the ER Ca^2+^ channel ryanodine receptor, and induction of ER stress [57, 208]. ER-resident proteins are particularly vulnerable to S-nitrosylation and as such a positive feedback mechanism would create a scenario whereby excessive RNS/ROS increasingly deactivates protective ER-resident chaperones such as PDI, prolonging UPR activation, leading to increases in ROS/RNS generation eventually resulting in cell death [31]. ER dysfunction due to excessive oxidative/nitrosative stress may, thus, lead to the S-nitrosylation of PDI in neurodegenerative disease [31]. However, PDI family members PDIA1, PDIA3, and PDIA4 can be S-nitrosylated independently of UPR induction [209]. Alternatively, PDI located at the cell surface may also promote production of SNO proteins. It has been previously suggested that extracellular SNO proteins may transfer NO to the cytoplasm via the reducing activity of cell surface PDI [181, 182]. According to this theory, reduced NO may readily penetrate the plasma membrane, leading to SNO production [128] (Figure 2). Hence, the formation of SNO-PDI results in the abrogation of the normally protective isomerase/chaperone activity of PDI, which may contribute to protein misfolding and production of SNO proteins. This suggests that SNO-PDI may be a common pathological mechanism contributing to neurodegenerative diseases.

## 10. S-Glutathionylation and PDI

A link between S-glutathionylated PDI and neurodegenerative disease has not yet been established [210]. However, cysteine residues in the a and a′ domains of PDI make it a potential target for S-glutathionylation [211].

**Figure 3:**
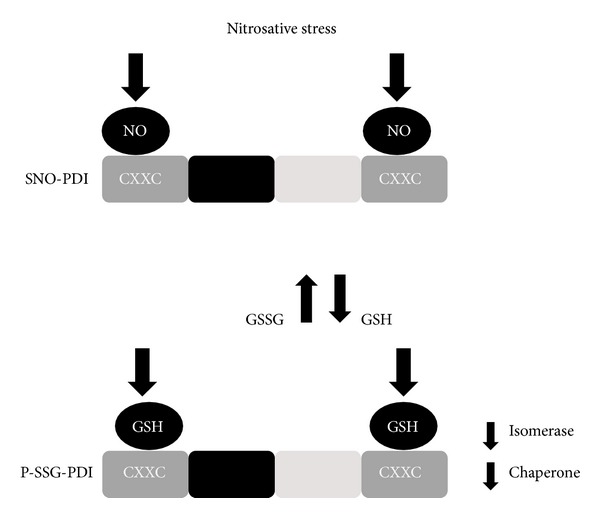
S-glutathionylation of PDI. Nitrosative stress from an exogenous agent (PABA/NO) increases intracellular NO and leads to the production of SNO-PDI. However, this may result in a decrease in GSSG/GSH ratio and increases in the free cellular pool of GSH. GSH then binds to the catalytic (a, a′) domains of PDI, resulting in S-glutathionylation (P-SSG) of its cysteine residues and attenuation of its protective isomerase and chaperone activity.

PDI has been shown to be S-glutathionylated at two of its four active cysteine sites (Cys53, Cys56 or Cys397, Cys400) [92]. S-glutathionylation was induced in these cells by treatment with anticancer agent O_2_–[2,4-dinitro-5-(*N*-methyl-*N*-4-carboxyphenylamino) phenyl]1–(*N*,*N* dimethylamino)diazen-1-ium-1,2-diolate (PABA/NO), which led to a dose-dependent increase in intracellular NO [210], triggering UPR induction and cell death [92]. S-glutathionylation of PDI has been demonstrated in human leukemia (HL60) and ovarian cancer cells (SKOV3) inhibiting its isomerase function [205]. In addition, S-glutathionylation of PDI abrogates its chaperone activity and prevents binding to oestrogen receptor alpha (ER*α*) [212]. The PDI-ER*α* interaction may protect ER*α* from oxidation and ensure its native protein conformation [213]. However, aberrant S-glutathionylation of PDI leads to destabilisation of the receptor and dysregulatation of ER*α* signaling. This may subsequently mediate cell death via activation of the UPR and reduced ER*α* stability [212]. However, although PABA/NO treatment increased levels of intracellular NO, it did not lead to S-nitrosylation of PDI [210]. There are two pools of S-nitrosylated proteins, GSH stable and GSH labile proteins, with the latter pool being readily subject to conversion to S-glutathionylated products [41]. Therefore, the lack of SNO proteins after PABA/NO treatment may be due to conversion of SNO proteins to S-glutathionylated proteins [210] (Figure 3). This notion therefore provides a link between S-nitrosylation and S-glutathionylation, although the exact relationship between these modifications remains unknown [41].

S-glutathionylation of PDI was proposed to be an upstream signalling event triggering misfolded protein accumulation and UPR induction [210, 211]. As PDI may regulate redox potential at the cell surface [182, 214], it therefore may facilitate cell adhesion [215], antigen processing [216], and glioma cell invasion [217]. S-glutathionylation of cell surface proteins alters extracellular and intracellular redox homeostasis [210]. Hence, irreversible S-glutathionylation/S-nitrosylation of cell surface PDI could alter redox potential, leading to amelioration of the protective chaperone/isomerase functions of PDI. This mechanism may therefore contribute to the excessive production of SNO and S-glutathionylated proteins observed in neurodegenerative disease. 

## 11. Conclusion

PDIs are a large family of chaperones and foldases which have complex yet still inadequately described functions with emerging roles in neurodegenerative diseases. Whilst S-nitrosylation plays a normal physiological role in signalling pathways, aberrant modification is triggered during conditions of elevated nitrosative and oxidative stress. Accumulating evidence suggests that SNO-PDI plays a role in the pathogenesis of neurodegenerative diseases such as AD, PD, and ALS, and this may exacerbate neurodegeneration via a number of mechanisms. However, most of the available reports are correlative in nature and therefore more direct approaches examining the contribution of S-nitrosylation of PDI family members to neurodegeneration are warranted. S-nitrosylation is also linked to another previously described modification of PDI, S-glutathionylation, although the S-glutathionylation of PDI and its role in neurodegenerative diseases have not been elucidated. Whilst PDI family members are conventionally regarded as being ER localized, they are also present and catalytically active in several other cellular locations, which is likely to be particularly important in disease as few proteins associated with neurodegeneration are found in the ER. Finally, cell surface PDI, which reduces NO allowing it to pass through the plasma membrane, may lead to the production of SNO proteins and therefore also contribute to the pathogenesis of neurodegenerative diseases. The broad involvement of PDIs in human neurodegenerative diseases highlights the need for a better understanding of how they become inactivated by posttranslational modification, which is crucial to evaluate their use as possible targets for disease intervention.
